# Socio-demographic and psychological factors associated with quality of life of women undergoing chemotherapy treatment for gynecological cancer

**DOI:** 10.1007/s00520-022-07162-8

**Published:** 2022-05-24

**Authors:** Valentina E. Di Mattei, Gaia Perego, Paola Taranto, Martina Mazzetti, Paola M. V. Rancoita, Francesca Milano, Giorgia Mangili, Emanuela Rabaiotti, Alice Bergamini, Raffaella Cioffi, Massimo Candiani

**Affiliations:** 1 School of Psychology, Vita-Salute San Raffaele University, Milan, Italy; 2 Clinical and Health Psychology Unit, IRCCS San Raffaele Scientific Institute, Milan, Italy; 3 Department of Psychology, University of Milano-Bicocca, Piazza dell’Ateneo Nuovo, 1, 20126 Milan, Italy; 4 University Centre for Statistics in the Biomedical Sciences (CUSSB), Vita-Salute San Raffaele University, Milan, Italy; 5 School of Medicine, Vita-Salute San Raffaele University, Milan, Italy; 6 Obstetrics and Gynecology Unit, IRCCS San Raffaele Scientific Institute, Milan, Italy

**Keywords:** Quality of life, Gynecological cancer, Chemotherapy, Oncology, Anxiety, Social support

## Abstract

**Purpose:**

This research aimed to investigate the socio-demographic, clinical, and psychological variables predictive of a greater functioning and quality of life in patients with gynecological cancer after their first cycle of carboplatin and taxol-based chemotherapy.

**Methods:**

The sample of the present research consisted of 104 patients. The European Organization on Research and Treatment of Cancer QLQ-C30, the State-Trait Anxiety Inventory-Form Y, and the Multidimensional Scale of Perceived Social Support were administered to each participant.

**Results:**

The analyses showed that higher state anxiety levels predicted a lower role, emotional, and social functioning and a lower general quality of life. Higher trait anxiety levels and social support perceived from one’s friends predicted a greater role functioning. Similarly, having a relationship predicted a greater physical, cognitive, and social functioning. On the contrary, the presence of relapsed cancer was negatively associated with these patients’ quality of life.

**Conclusions:**

The present study highlighted the importance of identifying patients at higher risk of experiencing lower levels of functioning and worse general quality of life to implement tailored interventions from the beginning of treatment, thus improving the quality of life of these patients throughout the chemotherapy treatment.

## Background

Gynecological cancers are among the most frequent cancers in the female population [1]. The diagnosis of cancer is an experience that forces patients to a profound and radical change not only in daily activities and life projects but also in their identity, role, responsibility, priorities, needs, and necessities [2]. Although advances in screening techniques and anticancer therapies have increased long-term survival, neoplastic disease and associated treatments still have numerous physical and psychosocial consequences that deeply affect patients’ quality of life [3–7]. The measurement of the health-related quality of life (HRQoL) in cancer patients includes the assessment of their subjective perception of symptoms, the side effects of treatments, and the consequences of the disease on various aspects of physical, role, emotional, cognitive, and social functioning [2, 8]. The assessment of HRQoL in oncology represents an important endpoint for clinical studies because there is a significant association between the overall quality of life, the domains of functioning, symptoms severity, adherence to treatments, and long-term survival [9–13].

Therefore, the identification of the factors associated with quality of life is of utmost importance.

Several studies identified the impact of clinical variables related to cancer and its treatment on quality of life, including physical symptoms (e.g., pain, fatigue, and emesis), neurocognitive disorders, sexual dysfunctions, and fertility loss [14–21]. In some cases, fertility loss due to cancer treatment can even be more devastating than cancer itself [19–22]. Other studies analyzed the predictive role of socio-demographic and psychological variables on cancer patients’ long-term quality of life. The literature shows that older age, low educational level, poor mental health, especially depression, poor perceived social support, low income, and unemployment status are risk factors for a lower long-term quality of life in different samples of cancer patients [23–30]. Fewer studies specifically investigated socio-demographic and psychological factors affecting the quality of life of cancer patients in the short-term, especially during chemotherapy. Although the current literature is scarce, it shows that high anxiety levels seem to be a risk factor for a lower short-term quality of life [31]. In contrast, high perceived social support, high educational level, and full-time employment (before and during therapy) appear to be protective factors and predictors of a better short-term quality of life [32–34]. Regarding age and marital status, the literature is unclear and shows contradictory results [32, 34, 35].

To identify patients who immediately need support and to improve their emotional wellbeing and long-term adherence to treatment, our study aimed to investigate which socio-demographic, clinical, and psychological variables may predict a better physical, cognitive, emotional, role, and social functioning and general quality of life, in patients with gynecological cancer after their first infusion of carboplatin and paclitaxel-based chemotherapy. In light of the aforementioned studies, we expect to find a protective role of social support, employment and low anxiety levels.

## Materials and methods

### Participants

The research, approved by the Ethics Committee of the IRCCS San Raffaele Hospital, was conducted on a sample of cancer patients under treatment in the Gynecology and Obstetrics Unit of the San Raffaele Scientific Institute in Milan.

Eligible women had to meet the following criteria: being at least 18 years old; having a gynecological cancer diagnosis; undergoing carboplatin and paclitaxel chemotherapy regimen (which is the standard chemotherapy for many gynecological malignancies); speaking and understanding Italian; having at least an elementary school certificate; and agreeing to voluntarily participate in the research by signing a written informed consent. Following these criteria, 105 women took part in the research. Patients were informed about the study by a psychologist during their first chemotherapy infusion.

Among these, 104 patients had no missing data in the EORTC QLQ-C30 functioning scales (physical, role, emotional, cognitive, and social) measured at the second chemotherapy infusion and were thus included in the analyses. Data were collected between February 2015 and November 2019.

### Measures

Patients completed a battery of tests during their first and second infusion of carboplatin and paclitaxel-based chemotherapy. During the first chemotherapy infusion, socio-demographic (i.e., age, presence of a relationship, presence of children, educational level, employment status, and intention to work after the chemotherapy infusion) and clinical information (i.e., presence of relapse) were collected from each patient using a specific questionnaire. The following questionnaires were also administered: the State-Trait Anxiety Inventory-Form Y (STAI-Y) and the Multidimensional Scale of Perceived Social Support (MSPSS). The European Organization on Research and Treatment of Cancer QLQ-C30 (EORTC QLQ-C30) was administered at the second chemotherapy infusion, to monitor the impact of the side effects of the first infusion of chemotherapy on quality of life.

The *State-Trait Anxiety Inventory*-Form Y (STAI-Y) [36] is a self-administered questionnaire of 40 items, on a 4-step Likert scale, that measures the severity of anxiety symptoms and differentiates state and trait anxiety. The state anxiety subscale evaluates the situational level of anxiety asking how respondents feel “right now, at this moment”; its items measure feelings of apprehension, tension, nervousness, worry, and arousal on a 4-step Likert scale (1 = not at all; 4 = very much). The trait anxiety subscale evaluates the relatively stable aspects of the “propensity/inclination to anxiety,” how the person feels and perceives oneself “generally,” including general states of calm, confidence, and security, measured on a 4-step Likert scale (1 = almost never; 4 = almost always) [32]. Scores for both subscales can range from a minimum of 20 to a maximum of 80. Cut-off scores have also been identified to differentiate patients with “low anxiety” (scores from 20 to 39), “medium anxiety” (scores 40 to 59), and “high anxiety” (scores from 60 to 80) [37]. The two subscales of the questionnaire show good reliability with Cronbach’s alpha coefficients ranging between 0.83 and 0.91 [36]. For the Italian version, the internal consistency coefficients for the state anxiety scale range from 0.91 to 0.95 (depending on the sample), and for the trait anxiety scale, the range is 0.85–0.90 [38].

The *Multidimensional Scale of Perceived Social Support* (MSPSS) [39] is a self-administered questionnaire of 12 items that evaluates the social support perceived by family, friends, and significant others using 4 items, on a Likert scale of 7 steps (1 = very strongly disagree; 7 = very strongly agree). The scores of each subscale and the total scale ranged from 1 to 7, with higher scores indicating higher perceived social support.

The internal reliability of the questionnaire is good, with the Cronbach’s alpha coefficient ranging from 0.85 to 0.91 [40]. The Italian version shows good indices of reliability with the Cronbach’s alpha coefficient ranging from 0.81 to 0.98 [41, 42].

The *European Organization on Research and Treatment of Cancer QLQ-C30* (EORTC QLQ C-30) [43] is a 30-item questionnaire composed of single-item and multi-item scales that investigates the construct of quality of life in cancer patients. The first 28 items have a response mode on a 4-step Likert scale and the last two on a 7-step Likert scale. The questionnaire measures the state of health over the previous 7 days through nine scales: five relating to functioning (“physical functioning,” “cognitive functioning,” “emotional functioning,” “role functioning,” “social functioning”), three relating to symptoms (“fatigue,” “pain,” “nausea/vomiting”), and one relating to the state of “general quality of life.” The questionnaire also includes six single-item scales that investigate the presence of symptoms and problems typically related to cancer and its treatment.

During the scoring, to facilitate interpretation, all the scores of the scales and the items are linearly transformed on a scale from 0 to 100. For the five scales of functioning and the general quality of life scale, higher scores represent higher levels of functioning and general quality of life. For the scales and items related to symptoms, higher scores correspond to greater symptom severity.

The questionnaire shows good reliability of multi-item scales with Cronbach’s alpha coefficient ranging from 0.54 to 0.86 before treatment and from 0.52 to 0.89 during treatment [43].

### Statistical analysis

The Wilcoxon test for paired data was used to compare the distributions of paired measurements. To assess the impact of socio-demographic, clinical and psychometric variables (the STAI-Y and the MSPSS) on the EORTC QLQ-C30 overall functioning, and quality of life subscales, each scale was categorized into high vs. low functioning, based on the median of the sample (greater than or equal to vs. less than the median, respectively). A multiple logistic regression analysis with a backward procedure of variable selection was conducted for each scale, considering all socio-demographic variables, the STAI-Y trait and state scales, and all MSPSS subscales (thus excluding the total MSPSS scale). A significance level of 5% was defined for all the analyses. All statistical analyses were carried out with the software MBI SPSS Statistics version 25 and R version 3.5.0.

## Results

Detailed descriptive statistics of the socio-demographic variables are reported in Table 1.

**Table 1:** Descriptive statistics of socio-demographic variables used in the analyses of functioning subscales and global quality of life

Variables	*N*	*n* (%)
In a relationship	103	
Yes	76	73.8%
No	27	26.2%
Children	104	
Yes	74	71.2%
No	30	28.8%
Bachelor’s degree	98	
Yes	22	22.4%
No	76	77.6%
Working after diagnosis	101	
Yes	24	23.8%
No	77	76.2%
Intention to work after 1st infusion	97	
Yes	22	22.7%
No	75	77.3%
Relapse	104	
Yes	23	22.1%
No	81	77.9%

The sample is composed of 104 patients with gynecological cancer aged between 27 and 83 years (median [interquartile range, IQR] = 58 years [50.00–67.00]). The type of cancer was ovarian for 71.1% of the patients (*n* = 74), endometrial for 22.1% (*n* = 23), cervical for 5.8% (*n* = 6), and uterus for 1% (*n* = 1); 22.1% of the sample (*n* = 23) have a relapsed cancer.

Most patients are in a stable relationship (73.8%, *n* = 76) and have children (71.2%, *n* = 74). Only 23.8% of patients were working after diagnosis (*n* = 24), and 22.7% of them declared willingness to work after the first chemotherapy infusion (*n* = 22, with 7 missing data).

Table 2 illustrates the median and interquartile range (IQR) for each subscale of the STAI-Y, the MSPSS, and each functional and global quality of life subscales of the EORTC QLQ-C30.

**Table 2:** Descriptive statistics of the psychometric variables

Variable	*N*	Median	IQR
STAI TRAIT	103	36.00	31.00–42.00
STAI STATE at 1st infusion	103	40.00	34.00–50.00
MPSS FAMILY	103	7.00	6.50–7.00
MPSS FRIENDS	103	7.00	5.25–7.00
MSPSS SIGNIFICANT OTHERS	103	7.00	6.75–7.00
MPSS TOTAL	103	6.67	6.00–7.00
Physical Functioning at 2nd infusion	104	93.33	80.00–100.00
Role Functioning at 2nd infusion	104	100.00	83.33–100.00
Emotional Functioning at 2nd infusion	104	83.33	66.67–91.67
Cognitive Functioning at 2nd infusion	104	100.00	83.33–100.00
Social Functioning at 2nd infusion	104	100.00	83.33–100.00
GLOBAL QUALITY OF LIFE	104	75.00	58.33–83.33

According to the cutoffs reported in the literature [37], most patients show low scores on the STAI-Y trait anxiety scale (median [IQR] = 36 [31.00–42.00]) and medium scores on the STAI-Y state anxiety scale (median [IQR] = 40 [34.00–50.00]). Moreover, at the first chemotherapy infusion, the median of the scores obtained on the state anxiety scale is significantly higher than the median of the scores obtained on the trait anxiety scale (*p* < 0.001 of the Wilcoxon paired test).

Regarding the MSPSS, most patients report good perceived social support in all three subscales with respect to the range of the scales (median = 7.00 for all three subscales, with the subscale MSPSS FRIENDS having the largest IQR [5.25–7.00]).

Finally, regarding the EORTC QLQ-C30 functioning subscales, most of the patients show a good level of functioning after the first chemotherapy infusion with respect to the range of the scales (physical functioning’s median = 93.33; role functioning’s median = 100; emotional functioning’s median = 83.33; cognitive functioning’s median = 100; social functioning’s median = 100). The median of the global quality of life subscale (median [IQR] = 75, [58.33–83.33]) is lower than the median of all functioning scales (for all, *p* < 0.001 of the Wilcoxon paired test), except for the emotional functioning (*p* = 0.002 of the Wilcoxon paired test).

In Table 3, all final multiple logistic regression models evaluating the effects of socio-demographic and psychometric variables on functioning subscales and global quality of life are reported. Multiple regression analyses show that higher state anxiety levels at the first infusion predict lower scores in role (OR = 0.911, *p* < 0.001), emotional (OR = 0.956, *p* = 0.017), and social functioning (OR = 0.932, *p* = 0.001) and a lower global quality of life (OR = 0.952, *p* = 0.012). Higher trait anxiety levels and social support perceived by friends predict higher scores in role functioning (OR = 1.071, *p* = 0.036; OR = 1.317, *p* = 0.034; respectively). Having a relationship predicts higher scores in physical (OR = 3.067, *p* = 0.017), cognitive (OR = 2.607, *p* = 0.038), and social functioning (OR = 3.505, *p* = 0.012). On the contrary, the presence of relapse is negatively associated with global quality of life (OR = 0.324, *p* = 0.032).

**Table 3:** Final models of multiple logistic regression analysis to predict high level of functioning subscales or global quality of life at the 2nd infusion

Variable	OR (CI 95%)	*p* value
Physical functioning		
In a relationship	3.067 (1.219–7.718)	0.017
Role functioning		
STAI TRAIT	1.071 (1.004–1.141)	0.036
STAI STATE at 1st infusion	0.911 (0.867–0.958)	< 0.001
MSPSS FRIENDS	1.317 (1.021–1.7)	0.034
Emotional functioning		
STAI STATE at 1st infusion	0.956 (0.921–0.992)	0.017
Cognitive functioning		
In a relationship	2.607 (1.053–6.453)	0.038
Social functioning		
In a relationship	3.505 (1.321–9.3)	0.012
STAI STATE at 1st infusion	0.932 (0.895–0.972)	0.001
Global quality of life		
Relapse	0.324 (0.116–0.909)	0.032
STAI STATE at 1st infusion	0.952 (0.915–0.989)	0.012

## Discussion

The purpose of the present study was to investigate which socio-demographic, clinical, and psychological variables may predict a better functioning, defined by the subscales of functioning and general quality of life of the EORTC QLQ-C30, in patients with gynecological cancer after their first infusion of carboplatin and paclitaxel-based chemotherapy.

As hypothesized, our findings show that high levels of perceived social support, low anxiety, and being in a relationship play a protective role on the quality of life of cancer patients during chemotherapy. In contrast to our expectations, we did not find a protective role either for being employed after the diagnosis or for intending to work after the first chemotherapy infusion.

Specifically, higher state anxiety levels at the first chemotherapy infusion predict a lower role, emotional, and social functioning and a lower global quality of life measured at the second chemotherapy infusion. Concerns and worries about chemotherapy outcome, side effects, and risk of relapse may affect the ability of patients to perform the usual social and domestic roles. Similarly, anxiety and fear of treatment and side effects have been found to increase patients’ overall stress levels and impair their emotional functioning [31]. The literature also identified social support by friends and younger age as predictors of better emotional functioning [34]. Moreover, increased anxiety and body image concerns, due to both cancer and chemotherapy, and fear and uncertainty about the future may negatively affect the relationship with family and friends, leading patients to avoid social moments [31].

Finally, higher levels of state anxiety at the first chemotherapy infusion negatively influence the global quality of life. This result is in line with the study of Charalambous et al. [31].

Our findings also show that higher trait anxiety and higher social support perceived by friends predict a higher role functioning. Considering that most women of the sample display low levels of trait anxiety (median = 36, IIQ = 31.00–42.00), it is possible that low levels of this stable personality feature allow patients to exercise more control in daily activities and to be more efficient in this area, despite the disease and its treatment. Moreover, a strong social network could offer more opportunities for recreation and fun, thus increasing role functioning.

Regarding role functioning, our findings seem to be in contrast with the literature. Specifically, working during chemotherapy [34] and being in a relationship [32] have been found to predict a higher role functioning. However, Goker et al. [32] evaluated role functioning 3 months after the end of the treatment, but not during chemotherapy. In addition, none of these studies took into account the levels of anxiety experienced by patients during chemotherapy.

Moreover, in the present study being in a relationship predicts higher levels of physical, cognitive, and social functioning. This result could be explained by the fact that being in a relationship frequently engages patients in interpersonal interactions, thus requiring a greater cognitive and physical involvement than patients who live alone. Therefore, the partner can be a source of support and motivation to be more active, both physically and mentally. In addition, the partner is a source of psychological and social support [33] and possibly increases the number and frequency of social interactions, thus facilitating a higher social functioning. However, a study conducted on a sample of patients with sarcoma did not find such associations [35]. Moreover, there could be cultural differences, as Goker and colleagues [32] reported that being married decreased the probability of higher social functioning in a sample of Turkish patients.

Finally, in our research, relapse is negatively associated with global quality of life, in line with the study by Wu et al. [44]. Indeed, the diagnosis of a relapsed disease has a devastating impact on patients’ lives, as they often experience a worsening of their physical condition and greater psychological distress with higher rates of depression, fear of death, and hopelessness, which significantly affect quality of life [45].

Some limitations of the present research must be acknowledged. First, the levels of functioning and the global quality of life were measured only after the first chemotherapy infusion; therefore, we cannot conclude that the variables found to be predictive of the functioning measured after the first infusion remain so throughout treatment. Moreover, levels of functioning could change during the chemotherapy infusions, as side effects may become more debilitating, patients may need more help for their daily activities, and distress levels could intensify and worsen.

Second, we did not consider some factors that could significantly affect the levels of functioning, such as the presence and severity of depressive symptoms and sexual functioning. Furthermore, we could not control for type of cancer as possible mediating or moderating variable in the analyses, due to the high unbalanced distribution of this variable in the sample which could affect the result of the analysis.

Despite these limitations, the main strength of this study is that our sample consists of patients with gynecological cancer undergoing the same chemotherapy regimen, thus reducing the heterogeneity of possible side effects.

## Conclusion

The present findings showed that being in a relationship, perceiving higher social support, and displaying lower anxiety levels at the first infusion of carboplatin and paclitaxel-based chemotherapy are the main protective factors for the levels of functioning and the global quality of life of patients with gynecological cancer after the first infusion.

These findings could help identify patients at higher risk of experiencing lower levels of functioning and a worse global quality of life to implement tailored interventions. Moreover, our findings clearly suggested the importance of providing support interventions from the beginning of treatment to decrease anxiety levels and improve these patients’ quality of life.

In the future, it could be interesting to analyze patients’ levels of functioning and quality of life up to the last chemotherapy infusion, to test whether the factors identified as significant in these analyses continue to play a predictive role throughout cancer treatment. In addition, it may be worthwhile to investigate couple relationships and sexual satisfaction of both patients and their partners. In fact, gynecological cancer is a type of cancer that uniquely affects sexuality, and both patients and their partners may experience high levels of emotional distress and sexual problems, which can have a negative impact on the quality of the relationship [19, 46].

## Data Availability

Data cannot be shared because participants did not provide written informed consent for it.
